# A school-based educational on-site vaccination intervention for adolescents in an urban area in Germany: feasibility and psychometric properties of instruments in a pilot study

**DOI:** 10.1186/s12889-021-12443-8

**Published:** 2022-01-10

**Authors:** Norma Bethke, Paul Gellert, Nina Knoll, Niklas Weber, Joachim Seybold

**Affiliations:** 1 Medical Directorate, Charité – Universitätsmedizin, Berlin, Germany; 2 Institute of Medical Sociology and Rehabilitation Science, Charité – Universitätsmedizin, Berlin, Germany; 3 Division of Health Psychology, Department of Education and Psychology, Freie Universität Berlin, Berlin, Germany; 4 Deutsche Gesellschaft für Internationale Zusammenarbeit (GIZ) GmbH, Bonn, Germany

**Keywords:** Vaccination, On-site, Mobile health, Prevention, School-based, Education, Measles

## Abstract

**Background:**

Vaccination rates for measles, mumps, and rubella (MMR) and diphtheria, tetanus, pertussis, and polio (Tdap-IPV) are not optimal among German adolescents. Education in combination with easy access to vaccination may be a promising approach to improve vaccination rates. The present paper describes a pilot study of a planned cluster randomized controlled trial (cRCT) in which we aim to improve MMR and Tdap-IPV vaccination rates together with knowledge and self-efficacy in a school setting.

**Methods:**

The study covered 863 students from 41 classes of four schools. The optimization and feasibility of access to schools, recruitment strategies, intervention, and assessment procedures were examined. The course and content of the educational unit were evaluated with a mixed-methods approach. A pre-post measurement design was tested for the vaccination rate in all schools. Additionally, at two schools, improvement in vaccination-related knowledge and perceived self-efficacy were measured by questionnaire pre-educational unit (*n*=287) and post-educational unit (*n*=293). The remaining two schools provided only postintervention data. Finally, we evaluated the psychometric properties (i.e., reliability, retest reliability, and change rates) of the questionnaire, applying Cronbach’s alpha, factor analyses, generalized estimating equations and linear mixed models.

**Results:**

The findings of the pilot study indicated good feasibility. Of the total sample, 437 students (50.9%) brought their vaccination cards to school, 68 students received Tdap-IPV vaccinations, and 11 received MMR vaccinations. Out of six knowledge questions, on average, the students had M=2.84 (95% CI [2.69, 3.10]) correct answers before and M=4.45 (95% CI [4.26, 4.64]) after the class. Ranging from 1 to 4, the self-efficacy scale changed by 0.3 points (p <.001); Cronbach’s alpha was 0.67 and 0.76 pre- and post-educational unit, respectively, and a one-factor solution was found. Content analysis of the five semistructured group interviews (n=12, 58.3% female) showed that all students found the length of the intervention to be appropriate. The teaching methods, including interactive and social media components, were perceived as very good.

**Conclusions:**

A school-based educational and on-site vaccination intervention appears to be feasible in terms of procedures and the adequacy of the instruments for the adolescent target group.

**Trial registration:**

ISRCTN, ISRCTN18026662. Pilot study for main trial registered 8 December 2017.

**Supplementary Information:**

The online version contains supplementary material available at 10.1186/s12889-021-12443-8.

## Background

With the Global Vaccine Action Plan (GVAP), the World Health Organization (WHO) set the global goal of increasing vaccination coverage together with eradicating and eliminating infectious diseases, including measles, polio, and pertussis, by 2020 [1]. From 2002 to 2018, the only region out of six WHO regions that could be considered consistently measles-free was the Americas [2]. However, even in this region, more than 10 countries reported endemic measles transmissions in both 2018 and 2019 [3]. For Germany, vaccination coverage for basic immunization and catch-up vaccination for mumps, measles, and rubella (MMR) and for tetanus, diphtheria, pertussis, and polio (Tdap-IPV) are too low and have been stagnating for years [4–6]. Measles outbreaks endanger the health of infected and nonvaccinated persons every year; 30% of infected persons develop one or more complications ranging from otitis media (7%) to pneumonia (6%), hospitalization (18%), encephalitis (0.1%), and even death (0.2%) [7]. In addition to individual health risks, measles represent an immense economic burden for the health system. Even for small measles outbreaks, which are defined as constituting fewer than 300 cases, the direct and indirect costs exceed the costs of a national vaccination program many times over with a benefit-cost ratio of 2.21 to 4.97 [8]. The cause of large pandemics is insufficient immunization rates [9]. Progress towards the GVAP global health targets is off track, and more efforts on a global level are required to address systemic weakness and limiting factors.

Effective approaches to increase vaccination rates are needed, and school-based on-site interventions may be feasible (for a narrative review of randomized controlled trials testing educational strategies in combination with on-site vaccination, see Additional file 1: Appendix 1: samples, intervention components, vaccinations carried out, vaccination process, outcome measures, and results). Whereas there is evidence that school-based interventions increase vaccination rates for influenza [10, 11] and sexually transmitted infections/human papillomavirus (STI/HPV [12–21]), school-based vaccinations against diseases such as measles, polio, and pertussis have only rarely been addressed within randomized controlled designs [11, 21, 22]. In interventions, common theoretical concepts that are assumed to drive the intervention effects include perceived barriers and benefits (e.g., as part of protection motivation theory [PMT] [23], social cognitive theory [SCT] [24, 25], and the health belief model [HBM] [26]) as well as perceived risk (e.g., PMT, HBM), all of which may be altered by knowledge and awareness strategies. Furthermore, a key determinant in most vaccination interventions is self-efficacy – individuals’ belief in their competency in goal-directed behavior (e.g., SCT, PMT). Moreover, environmental barriers and opportunities such as ease of access to vaccination and the direct offer of vaccination may increase the likelihood of action (e.g., HBM, SCT). This concept is captured as a “cue to action” in the HBM and is indirectly included in the SCT as environmental factors. However, cues to action are assumed to work in orchestration with self-efficacy beliefs, knowledge and outcome expectancies [26, 27].

Interventions that combine educational components with on-site vaccination appear to be a promising strategy for enhancing vaccination among adolescents (see Additional file 1: Appendix 1; [10, 11, 13, 18, 20, 21, 28–30]). Such studies show good feasibility and acceptability among staff and students, although comparability between studies is limited in terms of legal basis (e.g., national vaccination schedule, legal terms regarding vaccination consent of minors, who is allowed to carry out the vaccination), and they often incompletely report pretrial vaccination rates [11, 13, 20, 22, 28, 30].

Although there is growing evidence on the feasibility and effectiveness of school-based on-site vaccination interventions, more rigorous research is needed, combining theory-driven education and on-site opportunities for vaccination with objective assessments of effectiveness.

### Aims

The aims of the present pilot study were to test the feasibility of access strategies to schools, intervention procedures and the psychometric properties of the instruments used in a planned cluster randomized controlled trial (cRCT).

The planned cRCT was designed to identify effective educational strategies to increase vaccination rates for routine vaccines such as MMR or Tdap-IPV in students. For this planned cRCT, we developed an evidence- and theory-based, educational on-site vaccination intervention delivering vaccinations on school grounds in the Prevention Bus (see study protocol [31]), which was first applied and tested in the present pilot study.

Accordingly, this pilot study had three main objectives. The first aim was to test whether access to schools and recruitment strategies were suitable and effective for the planned cRCT. Second, the procedures of the educational/basic information intervention in combination with on-site, school-based vaccination were tested regarding feasibility. Third, measurement instruments including a self-report questionnaire on vaccination-related knowledge and a short version of a vaccination-related self-efficacy scale were evaluated in terms of internal consistency and sensitivity to change.

## Methods

### Design and study overview

**Fig. 1:**
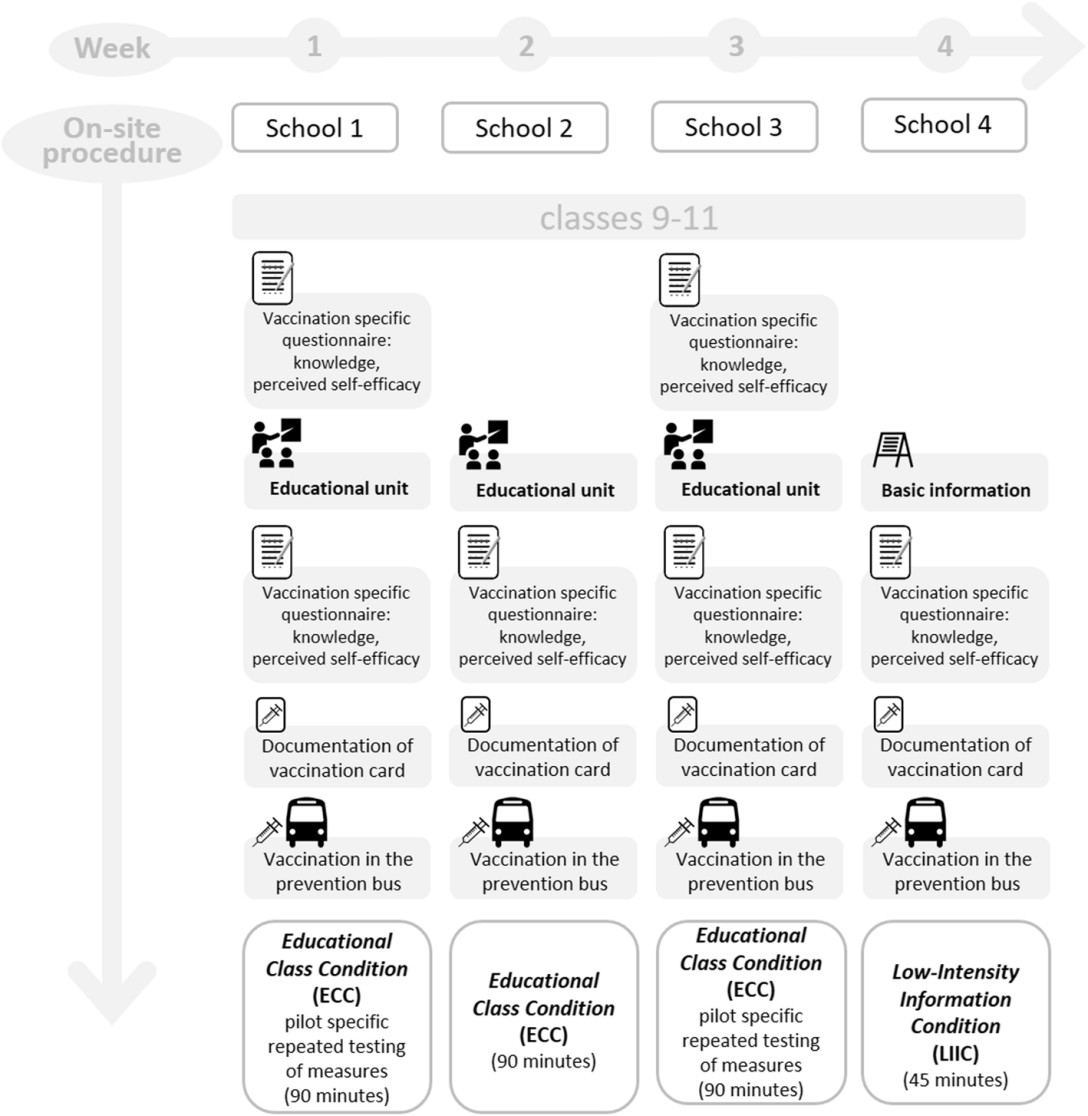
On-site procedures for specific schools, each carried out in the individual school class context

The pilot study was conducted using a four-group intervention design from August to September 2017. We applied a pre-post session design, adapted from the Solomon four-group design [32], at two schools and only a postsession design at two other schools. The procedure at each school is illustrated in Fig. 1.

In the pilot phase, the schools were not randomized to conditions. Design elements that were to be evaluated regarding feasibility were allocated at the school level. More detailed information on the intervention conditions can be found in brief in the 6 section and in more detail in the study protocol of the planned cRCT [31].

All four participating schools were visited by the Prevention Bus team and the bus for an entire school week. The Prevention Bus team on-site at each school consisted of a medical team, with two physicians and two nurses, and a bus driver. The bus was converted into a doctor’s office in 2016, enabling vaccinations to be carried out inside it directly on school grounds, fully meeting medical quality standards. A detailed description of the Prevention Bus can be found in the study protocol [31] and additional materials were included in the Additional file 1: Appendix 2 (a). Information material and consent documents were distributed to all students and their parents by the school staff one week in advance. During the week on-site at school, the students were addressed in their respective class contexts with either one or another intervention procedure (see Fig. 1) that differed with respect to the level of knowledge transfer and interactivity (see Methods, Intervention conditions). Both procedures ended with a joint visit of the class to the Prevention Bus and the opportunity to receive the MMR or Tdap-IPV vaccination. However, the vaccinations could also be carried out on any other day of the week while the bus and medical team were on-site.

### Study population

Schools in the pilot study were eligible for participation when they fulfilled two out of three inclusion criteria for the planned cRCT. For the pilot study, the recruited schools constituted a convenience sample. The inclusion criteria required that a school be an upper secondary school, i.e., a high school or a vocational school (for more information, see Additional file 1: Appendix 2 (b)) with at least 200 students in grades 9 to 11, and located in the city center of Berlin. This approach was chosen to achieve maximum comparability without interfering with schools eligible for the planned cRCT. Schools were recruited according to the described criteria, assuring balanced recruitment and the applicability of the pre-post design specific to the pilot study to different school types.

Students within the selected schools were eligible for participation in the educational/basic information components of the intervention if they were currently attending grades 9 to 11. The interventions were delivered during regular class times. Completing questionnaires and receiving vaccinations were optional.The vaccination decision for students under 18 years old required parental consent in the form of a signed consent form. For students under the age of 15, written parental consent was additionally reconfirmed by phone with the parent. For more information regarding the consent process, see Additional file 1: Appendix 2 (c).

### Intervention conditions

The development of the intervention components was theory- and evidence-driven. It is described in detail in the study protocol of the planned cRCT [31] in accordance with the template for the intervention description and replication (TIDieR [33]) checklist and guide (e.g., theories, procedures, training). The following is a brief description of the intervention conditions that were tested regarding feasibility [31].

At all four schools included in the study, the Prevention Bus was present for an entire school week. The school secretary’s office received take-home materials together with a declaration of consent form for the parents one week in advance. The school administration was instructed to distribute the materials and to remind the students 1-2 days in advance to bring the materials, including their vaccination card, with them on the day of the intervention.

Two planned intervention conditions were tested, i.e., the *Educational Class Condition* (ECC) and *Low-Intensity Information Condition* (LIIC). Both conditions took place in the school class context. The ECC/LIIC both included the completion of the questionnaires in the classroom and a guided tour of the Prevention Bus. Students from all participating schools had an opportunity to be vaccinated. See Fig. 1 for the on-site procedure for each school.

### ECC

At three schools (schools 1, 2 and 3), a 90-minute procedure was carried out that included an educational unit (45 min) taught by a physician in the classrooms. The educational unit consisted of a digital PowerPoint presentation with interactive elements. The topics addressed were the immune system and infectious diseases, vaccination processes, herd immunity, and the risks and benefits specific to measles vaccination. A group discussion as well as media elements such as newspaper articles and videos were also included. The educational unit was based on three theories of health behavior change (see Background, and study protocol of the planned cRCT [31]): SCT [24, 25], PMT [23], and the HBM [26]. It was intended to increase knowledge by providing and discussing information [23–26], to address vaccination-/disease-related risk perception by risk communication [23], and to use a testimonial of a fictional role model to address self-efficacy [24, 25].

### LIIC

In school 4, a 45-minute unit was conducted with the individual school classes. In each participating class, the physicians orally provided brief information about the Prevention Bus and the vaccinations offered. Detailed vaccination questions were not discussed within the LIIC. Solely procedural information and information already provided in the written vaccination consent form were addressed.

### Measures

With respect to the three aims, assessing the feasibility of the recruitment process and the feasibility of on-site procedures and to evaluate measurement instruments, we collected data for seven process indicators and measures: *school recruitment log*, *vaccination documents*, *vaccinations delivered*, *rating of the educational unit*, *semistructured interviews on the educational unit*, *vaccination-related knowledge scale*, and *vaccination-related perceived self-efficacy scale*. In addition, *covariates* that could influence vaccination behavior were assessed.

Using a *school recruitment log*, recruitment approaches and strategies were documented.

We assessed the presence and contents of *vaccination documents* as an indicator of the feasibility of on-site procedures regarding the requirement for vaccination in schools. We recorded the vaccination status (vaccinations: type and number of doses received) to determine the proportion of students with a need for vaccination in accordance with the German Vaccination Committee “Ständige Impfkommission am Robert-Koch Institut” (STIKO) [34]. All indicators were documented by a physician or nurse during the ECC/LIIC and before on-site vaccination was offered.

For the *vaccinations delivered* on-site, the medical team documented how many students received a vaccination in the Prevention Bus. In this study, combination vaccinations against mumps, measles, rubella (MMR [35]) and tetanus, diphtheria, pertussis and polio (Tdap-IPV [36])^1^ were offered. Vaccinations delivered were documented separately for MMR and Tdap-IPV at class-level.

The *rating of the educational unit* by all students in the ECC was documented. The students evaluated the course and content of the educational unit with a single item on a 3-point Likert-scale (scale: very interesting, interesting, or boring) administered after the ECC. Further feedback was recorded with *semistructured interviews in the educational unit* with selected students (single and group settings) in the ECC. The interviews were conducted after the students completed the individual questionnaire but before they were offered vaccinations in the bus. The translated questions can be found in Additional file 1: Appendix 2 (d).

**Fig. 2:**
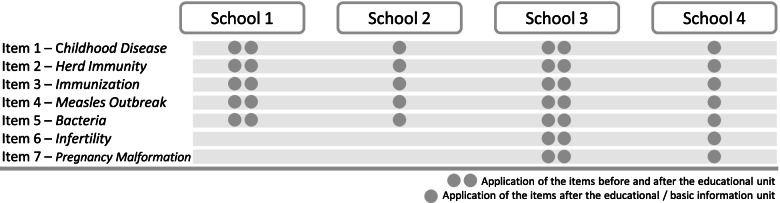
Vaccination-related knowledge items per school, including newly introduced items at schools 3 and 4

Using a maximum of seven items, the *vaccination-related knowledge scale* indicated the level of immunization knowledge (see Additional file 1: Appendix 2 (e)). Figure 2 provides an overview of which items were assessed at which school. The items covered facts about target groups of infectious diseases, prevention of the spread of infectious diseases, herd immunity, vaccination, and side effects of infections. The items were multiple choice, with one correct answer. Across all items, an overall knowledge sum score could be generated.

Two different sum scores were calculated in the pilot study. The 4-item sum score included items 1-4 (*childhood disease, herd immunity, immunization, measles outbreak*) and was assessed in all four schools (see Fig. 2). In addition, for schools 3 and 4, a 6-item sum score was computed as all final items were applied, including the newly successively introduced items 6 and 7 (*infertility, pregnancy malformation*). A low sum score (0) represented a low level of vaccination-related knowledge, and a high score (4 or 6) represented a high level of vaccination-related knowledge. Item 5 (*bacteria*) was not selected for the formation of scales. See Additional file 1: Appendix 2 (e) for information on item adaptations.

The v*accination-related perceived self-efficacy scale* reflected students’ beliefs about their own competency to understand, appraise, and apply vaccination-related health information (see Additional file 1: Appendix 2 (e)). The scale consisted of a short version of the European Health Literacy Survey Questionnaire (HLS-EU Q47,[37]). The short version was created by selecting items that covered vaccination and prevention. The students were asked to rate five items on a 4-point Likert-type scale ranging from 1 (very difficult) to 4 (very easy). A mean score was computed, with low scores representing low and high scores representing high levels of perceived self-efficacy.

Additionally, *covariates*, including gender, socioeconomic status (SES), and migration status, were assessed at the school and/or individual level. Regarding SES, two items were applied: whether students at home had a room of their own [38, 39] and whether they owned a “berlinpass”, a ticket that provides students of unemployed parents or parents with low income with access to reduced public transport fares and discounted leisure activities. Migration background was documented at the school level, indicating the ratio of students with a foreign-language background.

### Analysis

The data were analyzed using IBM SPSS Statistics 25. Descriptive data of the *school recruitment log* were used to evaluate the feasibility of the recruitment processes. Prior to the scale analyses, missing values for all final vaccination-related knowledge items (ranging from 2.2 to 7.3%) and all perceived self-efficacy items (ranging from 9.5 to 12.0%) were imputed with a multiple imputation (MI) procedure using fully conditional specifications and 20 imputed datasets.

As documented in the *school recruitment log*, a ratio between the targeted schools and successfully recruited schools was used. Furthermore, absolute numbers of *vaccination documents* brought to school (the number of vaccination cards present, consent forms returned) and *vaccinations delivered on-site* were reported. A relative ratio of students with a need for vaccination and vaccinations carried out in this study were reported at a descriptive level.

For the *vaccination-related perceived self-efficacy scale*, the internal consistency (i.e., Cronbach’s alpha) and the factorial structure (varimax-rotated exploratory factorial analysis [EFA]) separately for pre- and post-educational unit were computed. To determine significant changes from pre- to postintervention in *vaccination-related knowledge* and *perceived self-efficacy*, linear mixed models (LMMs) for continuous outcomes (covariance type=variance components, estimation=restricted maximum likelihood) and generalized estimating equations (GEEs) for dichotomous outcomes (distribution=binomial, link function=logit) were used; these approaches allowed for an adjustment for the nested structure of students in classes and controlled for the influence of age, gender, and SES. The longitudinal pre- and post-educational unit measurements of the questionnaire in the same school class (see Fig. 1, schools 1 and 3) were used to test sensitivity to changes in vaccination-related knowledge and perceived self-efficacy measurements. In addition, we compared the schools in which the questionnaire was collected only once, either post-educational unit (school 2) or post-basic information (school 4).

All tests of significance were based on the *p* < .05 level, and confidence intervals of 95% were achieved.

To evaluate the feasibility of the educational unit, the frequencies of student ratings of the educational unit were used. Furthermore, a research assistant coded the transcripts of the semistructured interviews, and interrater reliability was tested by another research assistant coding the transcript. The mean-level results and distributions are presented at a descriptive level.

## Results

### Sample Characteristics

For this pilot study, four schools were contacted, and all four agreed to participate. In addition to prior email contact, preparatory telephone calls were made to all schools to provide additional information. At one school, an additional appointment was made in advance because more detailed information regarding on-site procedures was requested. One junior high school, one junior-senior high school and two academic high schools (see Additional file 1: Appendix 2 (b)) constituted the pilot sample. School 1 was located in the city center, and schools 2, 3, and 4 were located in a district of Berlin adjacent to the city center. The four enrolled schools had a total of 41 school classes in grades 9 to 11 and 863 participating students, with an average age of 14.8 years; 49.4% were female (see Table 1).

**Table 1:** General characteristics of schools and students included in the pilot study

	Total	School 1 *ECC*	School 2 *ECC*	School 3 *ECC*	School 4 *LIIC*
No. of days present at school	18	5	5	3	5
School type	Public schools	Junior high school	Junior-senior high school	Academic high school	Academic high school
No. of classes	41	8	13	6	14
No. of students	863	161	292	130	280
Ø no. students/class	21.0	20.0	22.5	21.2	20.0
Ø age in years (SD in years)	14.8 (1.0)	14.8 (0.8)	14.9 (1.0)	14.6 (1.0)	14.9 (1.0)
Female, %	49.4	41.5	48.5	50.4	54.3
Ø Household size – people (SD)	4.2 (1.5)	4.7 (1.7)	4.3 (1.5)	4.2 (1.5)	3.8 (1.2)
Room, yes in %	74.8	52.8	75.3	74.4	87.2
Low SES, %	35.3	65.8	40.7	27.3	15.2
Foreign-language background, %	47.2	87.9	48.0	45.2	23.8

*ECC* Educational Class Condition, *LCC *Low-Intensity Information Condition

### Vaccination Status

Out of the total sample, 437 students (50.6%) brought their vaccination card to school (see Table 2). The check of the vaccination cards yielded the possibility of identifying the vaccination status. This included how many doses of a vaccine someone had received and whether there was an indication/need for vaccination according to the official vaccination recommendations (STIKO).

The check of vaccination cards indicated that 16 students (3.7%) showed a need for the MMR vaccination. In the past, these students had received either only one vaccination or no vaccination against mumps, measles and/or rubella. Of these 16 students with a need for vaccination, 11 (68.8%) were vaccinated on-site as part of this study.

For Tdap-IPV vaccination, the check of vaccination cards indicated that 36 students (8.2%) had received only four doses of a tetanus, diphtheria, and pertussis vaccination (basic immunization to the age of 14 months) in the past and had a definite need for a booster vaccination. Another 152 students (34.8%) had received five doses of tetanus, diphtheria, and/or pertussis. Students with five doses showed a conditional need for a booster vaccination, and a sixth shot is recommended if the time distance to the last vaccination is greater than five years. Regarding polio, only 148 students (33.9%) had received up to four doses of a polio vaccination and had a need for a single booster with Tdap-IPV. In this pilot study, 68 students received Tdap-IPV vaccinations. It was not possible to provide information on the relative ratio of vaccinated students to the need for Tdap-IPV vaccination regarding tetanus, diphtheria, and pertussis. For these infectious diseases, five years should pass between vaccination doses five and six. For the students with five vaccinations, the time span to the last vaccination was not documented in the pilot study.

**Table 2:** Vaccination status for students with vaccination cards present and number of doses delivered on-site

Vaccination status for students with vaccination card present *N* = 437 (50.6% of the total sample)	Before intervention								Vaccine doses delivered on-site
	**0 doses** ^**b**^		**1 dose** ^**b**^		**2 doses** ^**d**^		**>2 doses** ^**d**^		**MMR**, *n*
	Basic immunization to age 2 years (23 months), 2 doses ^a^								
**Mumps**, *n* (%)	3 (0.7)		13 (3.0)		419 (95.9)		2 (0.4)		11
**Measles**, *n* (%)	2 (0.4)		12 (2.8)		418 (95.7)		5 (1.1)		
**Rubella**, *n* (%)	3 (0.7)		13 (3.0)		418 (95.7)		3 (0.7)		
	**0-1 doses** ^**b**^	**2-3 doses** ^**b**^		**4 doses** ^**b**^		**5 doses** ^**c**^		**6 doses** ^**d**^	**Tdap-IPV**, *n*
	Basic immunization to age 1.3 years (14 months), 4 doses ^a^					Booster immunization every 5-10 years ^a^			
**Tetanus**, *n* (%)	10 (2.3)	7 (1.6)		19 (4.4)		150 (34.3)		251 (57.4)	63
**Diphtheria**, *n* (%)	10 (2.3)	7 (1.6)		19 (4.4)		151 (34.5)		250 (57.2)	
**Pertussis**, *n* (%)	13 (3.0)	7 (1.6)		18 (4.1)		152 (34.8)		247 (56.5)	
	Basic immunization to age 1.3 years (14 months), 4 doses ^a^					1 booster immunization at age 9-14 years ^a^			
**Polio**, *n* (%)	14 (3.2)	13 (3.0)		121 (27.7)		269 (61.6)		20 (4.5)	

^a^ Vaccination recommendation of the German Vaccination Committee (Ständige Impfkommission am Robert Koch-Institut, STIKO [32]); ^b^ indication for a vaccination; ^c^ conditional indication for a vaccination for tetanus, diphtheria and/or pertussis, depending on time lag to previous vaccination; ^d^ no indication for a vaccination

The documentation for the vaccination documents (vaccination card, parental consent) was expanded during the course of this pilot study to take into account on-site feedback from physicians regarding parental consent/dissent (explicit objection) to vaccination. Originally, parental consent was recorded only for minors who received a vaccination. Furthermore, each minor also had to agree to the vaccination. However, some parents explicitly objected to the on-site vaccination. At schools 3 and 4 (*n*=297), 121 students (40.7%) presented their vaccination card, and 78 (64.5%) of them also had written parental consent. However, 9 (3.0%) of these students had a written objection from their parents against the vaccination. Fifteen students (5.1%) without a vaccination card nevertheless had parental consent with them. A further 23 students (7.7%) who did not have a vaccination card with them had a written objection from their parents opposing vaccination.

### Knowledge Scale

Concerning the vaccination-related knowledge items with repeated measures (ECC: pre- and post-educational unit, schools 1 and 3), when GEE models were applied to each item, the percentage of correct responses for the seven knowledge items before the educational unit ranged from 22% (CI 16%/28%) to 70% (CI 65%/76%). After the educational unit, these rates increased to 32% (CI 23%/40%) to 86% (CI 81%/92%) (Table 3).

Item 5 (*bacteria*; see Fig. 2) was excluded from all further analyses and thus was also not included in the formation of the knowledge sum scores. Students had reported that item 5 was difficult to understand, and the item showed no sensitivity to change from pre- to post-educational unit (see Table 3).

**Table 3:** Number of correct answers for the vaccination-related knowledge items with repeated measures

Measure	Knowledge Items (schools 1 and 3) Generalized estimated equations for each item (imputed knowledge items, covariates: age, gender, SES)			
Condition	Pre-educational unit		Post-educational unit	
	*% correct responses (CI)*	*N* students, *classes, schools*	*% correct responses (CI)*	*N* students, *classes, schools*
ITEM 1 *Childhood disease*	22 (16-28)	287, 14, 2	44 (38-50)	291, 14, 2
ITEM 2 *Herd immunity*	39 (32-46)	287, 14, 2	55 (44-66)	291, 14, 2
ITEM 3 *Immunization*	70 (65-76)	287, 14, 2	80 (76-84)	291, 14, 2
ITEM 4 *Measles outbreak*	61 (54-69)	287, 14, 2	86 (81-90)	291, 14, 2
ITEM 5 *Bacteria* ^a^	34 (30-39)	258, 14, 2	32 (23-40)	283, 14, 2
ITEM 6 *Infertility*	35 (24-46)	127, 6, 1	82 (78-86)	130, 6, 1
ITEM 7 *Pregnancy malformation*	37 (27-46)	127, 6, 1	68 (61-75)	130, 6, 1

^a^ Item 5 – Bacteria was excluded in the process of item selection; accordingly, it was not imputed, as only items used for the computation of the knowledge scale were imputed. Thus, item 5 is reported with missing responses (6.4%), and listwise deletion is applied

Based on the results displayed for every knowledge item, the final knowledge items were selected, and 4- and 6-item sum scores were computed. All scale analyses were performed with LMM, which enabled us to account for between-class and between-school variation as well as the class-level male/female ratio, age, and SES.

#### Knowledge sum score, change from pre- to post-educational unit

Concerning the 4-item knowledge sum score, for schools 1 and 3, there were two data points for each student. In these schools, the students worked on the questionnaire both pre- and post-educational unit (i.e., 578 data points, 2 schools, 14 classes, 2 occasions, mean age 14.6 years, 48.0% female). The LMM showed a significant change (B=-0.69, CI -0.86/-0.53, p <.001) from pre-educational unit (M=1.92. CI 1.76/2.08) to post-educational unit (M=2.61, CI 2.46/2.77).

The pre-post analysis with the 6 final knowledge items (i.e., 6-item knowledge sum score) included 127/130 students from 1 school (6 classes, mean age 14.6 years, 50% female). The LMM yielded a significant change (B=-1.55, CI -1.83/-1.27, p <.001) from pre-educational unit (M=2.84, CI 2.69/3.10) to post-educational unit (M=4.45, CI 4.26/4.64).

**Fig. 3:**
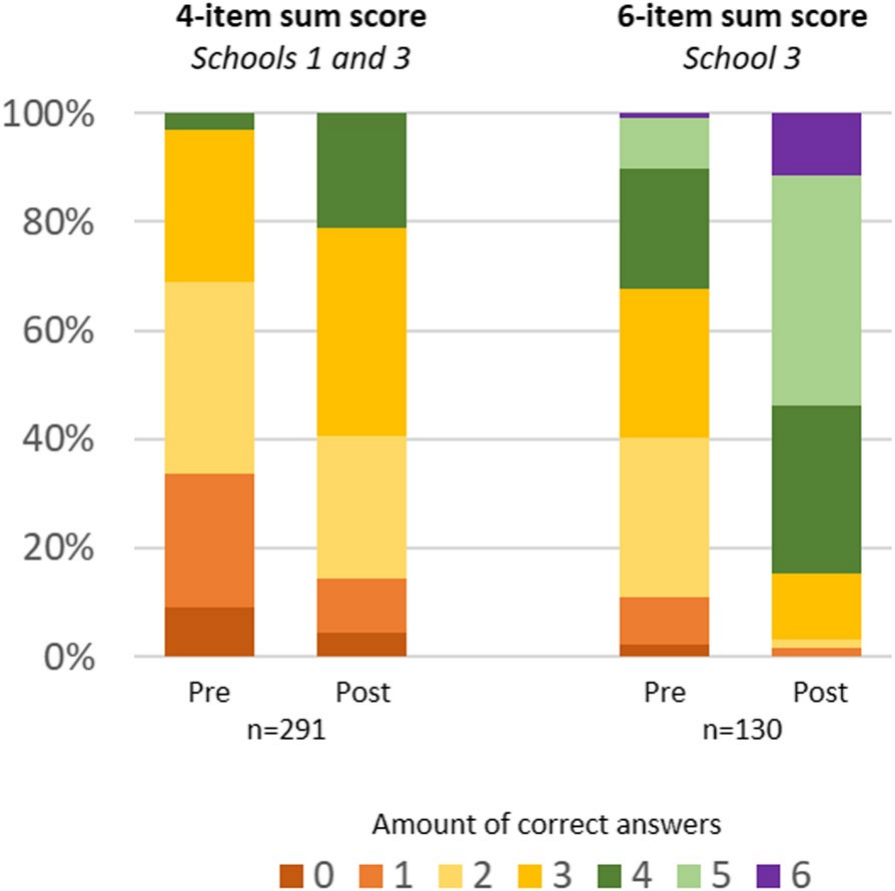
Distribution of the correct answers of the knowledge items for students with two data points

The distribution of the number of correct answers (4-item/6-item sum score) for the students included in the pre-post condition is shown in Fig. 3.

#### Knowledge sum score post-basic information versus post-educational unit

Furthermore, the distribution of the correct answers was compared for the 4-item sum score between two schools where the questionnaire was applied only once. In school 2, the questionnaire was administered post-educational unit (292 students, 13 classes, mean age 14.9 years, 48.5% female), and in school 4, it was administered post-basic information unit (280 students, 14 classes, mean age 14.9 years, 54.3% female). The LMM showed a significant difference (B=-0.64, CI -0.94/-0.33, p <.001), indicating a higher knowledge sum score post-educational unit (M=2.98, CI 2.78/3.17) than post-basic information (M=2.34, CI 2.15/2.54).

### Self-efficacy Scale

For perceived self-efficacy, the item means ranged between M=2.5 (CI 2.4/2.6) and M=3.4 (CI 3.3/3.5) post-basic information and between M=2.9 (CI 2.8/3.0) and M=3.5 (CI 3.5/3.6) post-educational unit (Table 4).

**Table 4:** Means (range: 1-4) of perceived self-efficacy items with repeated measures

Measure	Perceived self-efficacy items (schools 1 and 3) Linear mixed models for each item (imputed perceived self-efficacy items, covariates: age, gender, SES)			
Condition	Pre-educational unit		Post-educational unit	
	*Mean* *(CI)*	*N* students, *classes, schools*	*Mean* *(CI)*	*N* students, *classes, schools*
ITEM 1 *Why do I need vaccinations*	3.4 (3.3–3.5)	287, 14, 2	3.5 (3.5–3.6)	291, 14, 2
ITEM 2 *Which vaccinations do I need*	2.7 (2.6–2.8)	287, 14, 2	3.2 (3.1–3.3)	291, 14, 2
ITEM 3 *Trustworthiness of information (media)*	2.5 (2.4–2.6)	287, 14, 2	2.9 (2.8–3.0)	291, 14, 2
ITEM 4 *Advice from friends and family*	3.0 (2.9–3.2)	287, 14, 2	3.1 (3.0–3.3)	291, 14, 2
ITEM 5 *Advice from media*	2.7 (2.6–2.9)	287, 14, 2	3.0 (2.9–3.2)	291, 14, 2

The internal consistencies of the self-efficacy scale were alpha = 0.67 pre-educational unit and alpha = 0.76 post-educational unit, indicating acceptable reliability of the self-efficacy scale. When the factorial structure of the self-efficacy scale pre- and post-educational unit was explored by means of EFA, a unidimensional factor solution was suggested by the scree plots and the factor loadings in the component matrix were comparable for the items before and after the class.

Using all available data from students who completed the questionnaire pre- and post-educational unit (578 data points, 2 schools, 14 classes, 2 occasions; mean age 14.6 years, 48.0% female), an LMM that accounted for between-class variation was applied. The model showed a significant change of 0.3 points pre- to post-educational unit (B=-0.20, CI -0.40/-0.20, p <.001), with an increase from 2.9 (CI 2.78/3.00) to 3.2 (CI 3.10/3.24) on the perceived self-efficacy scale (range from 1 to 4).

Furthermore, we compared data from the other two schools, where the students worked on the questionnaire only once, either post-educational unit (school 2, 292 students, 13 classes, mean age 14.9 years, 48.5% female) or post-basic information (280 students, 14 classes, mean age 14.9 years. 54.3% female). The analysis revealed comparable results, showing a significant difference (B=-0.26, CI -0.33/-0.07, p <.01) for items assessed post-basic information (M=2.96, CI 2.88/3.04) and post-educational unit (M=3.15, CI 3.07/3.24).

### Educational Unit

The content analysis of the five semistructured interviews (*n*=12, 58.3% female) showed that all students considered the length of the educational unit to be appropriate. The teaching methods of the physicians were perceived as very good, especially regarding the activation elements and the possibility of interaction between the students and physicians. The social media post was rated as good or very good by four out of five groups. The content was considered modern, realistic, and easy to understand. The contents of the educational unit on the individual diseases as well as social aspects (e.g., herd immunity) and the video materials were perceived to be particularly interesting. The students stated that their attitude towards vaccination had not changed. Nevertheless, the ECC was perceived as a positive learning opportunity. Additional information on the diseases described during the ECC (e.g., on effects of rubella during pregnancy and on HPV) was requested by three groups.

## Discussion

The aims of the present pilot study were to test the feasibility of recruitment and on-site procedures (i.e., intervention conditions and on-site vaccination) of a planned cRCT. The further, methodological aim of this study was to evaluate the psychometric properties of the instruments.

The results of the current pilot study showed that the school-based on-site vaccination approach is a feasible way to improve vaccination rates. The recruitment strategies proved successful in gaining access to schools comparable to those that would be eligible for the planned cRCT. Students in all participating schools showed a good vaccination uptake rate after the pilot intervention and provided good feedback regarding the educational components of the intervention. Regarding the instruments’ psychometric properties, our measurement tool for vaccination-related knowledge showed a reasonable sensitivity to change. In addition, the self-efficacy scale presented with a one-factor structure was internally consistent, and self-efficacy levels regarding vaccination increased from before to after the educational unit.

For the successful implementation of the planned cRCT, the pilot study showed that our recruitment procedures for schools to participate in an educational, on-site vaccination project are very promising. In comparison to other on-site vaccination interventions, we successfully included 100% of the contacted schools, which represents an above-average participation rate [10, 13, 28–30]. To achieve an effective objective assessment of vaccination rates, it was also indispensable to gain a high participation rate of students providing their vaccination documents (i.e., vaccination cards and signed consent forms). Half of the students enrolled in this pilot study presented their vaccination cards. The parental consent rate for minors, documented at schools 3 and 4, was approximately 65%. The present participation rates thus corresponded to those of other on-site vaccination studies [11, 13, 18, 22, 28, 29]. Notably, documentation of response rate is not reported at all or very inconsistently in most studies (see Additional file 1: Appendix 1). The information that could be extracted from the studies indicated a range of number of vaccination documents brought to school and/or parental consent from less than 30% [11, 28], to 50% [22], up to 80% [13, 18, 29]. Thus, compared to the studies, the rate in our pilot study is in the comparable middle range. However, further efforts should definitely be made to improve the return rate of the vaccination documents. Suggested measures, which are based on other studies and feedback from the present participants, are a closer connection of teachers [29] and parents to the organization and the increased promotion of opportunities of contacting the study team, as well as improving the infrastructure to provide vaccination documents get vaccinated on subsequent days on the bus. Regarding vaccinations carried out, 11 out of 16 students, approximately 69%, with a need for vaccination received the MMR vaccination, and a total of 63 Tdap-IPV vaccine doses were administered. To be able to draw a conclusion about the total number of students who needed a booster vaccination, the time since the last Tdap IPV vaccination received must be determined. Due to insufficient information about the time passed since the last Tdap-IPV dose received, as there should be at least 5 years between doses five and six [34], the relative rate for this vaccination could not be determined. In sum, it is encouraging that the individual protection of the MMR vaccination in this study increased strongly. The need for the Tdap-IPV vaccination seemed to be relatively high, as approximately 15% of all students with a vaccination card had been vaccinated. Nevertheless, the documentation should be expanded. Generally, the applied recruitment processes and the on-site vaccination procedure seem to be feasible procedures for the planned cRCT.

As a result of the pilot study, we will improve documentation procedures on-site. For example, for the Tdap-IPV vaccination, in addition to the number of doses received, the time interval to the last Tdap-IPV vaccination should be recorded in the planned cRCT from the beginning to determine whether, after completing basic immunization (4 doses), a booster vaccination is due [34]. Without this information, the actual need for vaccination cannot be determined. Further, by additionally documenting written parental objection to vaccination, insightful important information was provided by the pilot study that should be included in the subsequent study. Parents were informed in advance that if they did not want a vaccination to be carried out, not signing the consent form was sufficient action. The number of unsigned consent forms can be used as an indicator of whether there is an increased need for further interaction with parents in general or with specific subgroups. In the pilot study, the documentation of parental objection was carried out for two schools, and the number was relatively low, with nine objections (3.0%). Whether there is a need for further interaction with the parents cannot be adequately evaluated on the basis of this pilot study because the informational value was quite limited due to the small number of schools and their characteristics. Both where we documented the parental objection were academic high schools with above-average SES. In the main study, the data on explicit objection to vaccination additionally should be further investigated to determine whether, for example, there are associations between SES or migration background and vaccination objection or consent. In combination with these indicators, vaccination laggards should also be recorded in the future. In this pilot study, students were defined as vaccination laggards if they were not vaccinated on the bus on the day of the intervention but were vaccinated later in the week. In this way, it is possible to determine whether a visit of several days to a school and for which school types this approach makes sense. Also in this context, in the main trial information of vaccination laggards should also be analyzed as to whether other students in the class have already been vaccinated. This can be discussed as an indicator for actual vaccination behavior and the importance of having a role model [24]. Additionally, it is useful to determine to what extent students from intervention groups use the educational components as a cue to action by taking delayed advantage of the on-site vaccination offer. The decision to use anonymous responses to the questionnaires and to document vaccination rates at class level was made explicitly in advance by the study team. This was the product of prior consultation with the schools and possible fears that parents and students might perceive the questionnaire responses as an individual knowledge test and that individual vaccinations or not receiving a vaccination could be traced back to the students. The pilot phase showed that this approach had a positive impact on the willingness to participate from schools, parents and students alike, as this point was repeatedly asked for. Broader documentation of data on vaccination needs, the parental consent process, and the time of take-up of vaccinations should be used in the future to increase the significance of a study.

Testing the previously developed theory- and evidence-based educational unit yielded helpful insights regarding which topics seemed to be of greater relevance to the students. According to the results of the qualitative interviews, for the planned cRCT, we slightly adapted the timing of the contents of the educational unit. This adaptation will result in a stronger focus on infectious diseases and social aspects. Length, thematic focus and interactive content as well as the implementation by a physician were considered positive by the majority of the students and could thus be adopted for the planned study.

The improvement in vaccination-related knowledge [12, 13, 15, 28] and the increase in perceived self-efficacy [12, 14, 18] are in line with previous findings. In terms of the knowledge scale, one item had to be dropped because of problems with understanding and lack of sensitivity to change. Two further items were introduced, both of which addressed complications of infectious diseases.

The findings of this pilot study indicate that it seems possible to increase the vaccination rate for mumps, measles, and rubella as well as tetanus, diphtheria, pertussis, and polio together with vaccination-related knowledge and vaccination-related self-efficacy in students.

### Strengths and limitations

The key strengths of our pilot study include the large sample size and the elaborate study design. The design allowed us to test the feasibility and properties under conditions comparable to those of the planned cRCT; furthermore, the design provided opportunities for adjustments and for comparisons of different assessment strategies (e.g., pre-post, post only with and without educational unit).

There are several limitations to this study. First, due to the small number of schools, we were not able to differentiate the feasibility and psychometric properties of the assessment tools on the basis of school type. However, in the planned cRCT, we will be able to account for these variations. For logistical reasons, migration status at the individual level [40] and a scale assessing SES, including information on the parents [41], could not be evaluated, but such an evaluation will be possible in the planned cRCT. More detailed information on SES and migration status on an individual level is also important, as studies have shown that vaccination interventions as well as educational interventions vary in their effectiveness as a function of migration status [42, 43]. Finally, although we accounted for confounders statistically, we did not allocate the schools randomly; thus, full control over confounders was not possible, and the results may be biased. The generalizability of the results in terms of change in vaccination coverage is not given here. However, the purpose of this pilot study was to attain insights into the feasibility of access strategies to schools, intervention procedures and psychometric properties, for which the current study design was appropriate. On-site vaccination at schools was also successfully feasible but should be further investigated in a randomized design.

### Implications for research, policy and practice

This pilot study has direct implications for the planned main study, particularly with respect to the reliability of the secondary outcome measures (e.g., vaccination-related knowledge) and concerning recruitment and data-handling structures. Furthermore, we showed substantial vaccination rates as well as rates of change regarding increases in knowledge and self-efficacy attributed to the educational unit. Moreover, future research may use the same assessments in other vaccination-related studies, either interventional or observational. We provided findings for the MMR and Tdap-IPV vaccinations; however, future research should include other types of vaccination, such as STI/HPV vaccinations, in the school-based approach [12–20]. These findings may also play a role in the context of the COVID-19 pandemic in terms of vaccinating students and staff directly in the school context. Especially with the possibility of regular booster vaccinations (e.g., annually), the Prevention Bus represents an efficient vaccination concept. Furthermore, we developed our intervention and procedures in the capital city of a high-income country. Future studies should test comparable approaches in low- and middle-income countries and in more rural areas, as the effects may be even larger in these regions.

Providing an on-site school-based vaccination program in combination with a health education intervention proved to be feasible and effective. The intervention may simultaneously increase the health literacy levels of adolescents and lower the structural barriers to vaccination. Nevertheless, for other future randomized controlled vaccination trial designs, the affective and implicit/impulsive aspects of vaccination decisions in comparison group designs should be addressed. Nudge theory [44] and dual process theory [45] suggest that emotional components, in particular, play a potential role in the school immunization context. Furthermore, this intervention strategy may be cost-effective and scalable and thus constitutes an additional public health strategy that might complement existing endeavors to improve vaccination rates. Data from the planned cRCT can be used to estimate cost-effectiveness and scalability. Subsequently, both the ECC and the LIIC should be reviewed in more detail with regard to a cost-benefit analysis to be able to represent the basis for practical care models of health policy.

## Conclusions

We found that a school-based educational and on-site vaccination intervention was feasible regarding recruitment strategies and on-site procedures, and the vaccination-related instruments showed adequate psychometric properties. Further pursuit of this line of research may improve the currently suboptimal vaccination rates and thus help to protect adolescents and people of different ages from infectious diseases.

## Supplementary Information

**Additional file 1.**

## Data Availability

The datasets generated and analyzed during the current study are not publicly available for participants’ confidentiality but are available from the corresponding author on reasonable request.

## Abbreviations

cRCT: cluster randomized controlled trial
EFA: varimax-rotated exploratory factorial analysis
LMM: linear mixed model
MI: multiple imputation
MMR: measles, mumps, rubella
Tdap-IPV: tetanus, diphtheria, pertussis, polio
OECD: Organisation for Economic Co-operation and Development
SES: socioeconomic status
WHO: World Health Organization

## Footnotes

1 We applied a quadruple booster vaccination against tetanus, diphtheria and acellular pertussis (Tdap) together with polio (IPV). The vaccine offered is comparable to vaccinations applied in other studies named DT, DTP, DTaP, Tdap, but varies in antigen content (small vs. capital letters), and included components (polio, pertussis not always included).
