# Supplementary material for: A school-based educational on-site vaccination intervention for adolescents in an urban area in Germany: feasibility and psychometric properties of instruments in a pilot study
Source: BMC Public Health. 2022 Jan 10;22:60. doi: 10.1186/s12889-021-12443-8 (PMC8744022; doi:10.1186/s12889-021-12443-8)
Supplement: Supplementary file 1 — Additional file 1. [file 12889_2021_12443_MOESM1_ESM.docx]

**Additional file 1**

## Appendix 1

**Table 1** Literature overview: School-based vaccination interventions for adolescents

| Study | Sample and randomization,  for longitudinal survey, data collection intervals | Intervention (IG=intervention group, CG=control group) | Recruitment procedure | Vaccination process | Eligibility for study participation and vaccination (parental consent) | Vaccinations delivered | Outcome measures and results |
| --- | --- | --- | --- | --- | --- | --- | --- |
| Daley et al. [11] | USA, Denver, urban public school district,  14 schools*, grade 6-8,  N=3,144  Cluster-randomized controlled trial, randomization at school level (7 schools IG & CG each),  Stratified (by 6^th^ vs. 7^th^-8^th^ grade and by percent of students qualifying for free/reduced lunch >70% vs. ≤70%).  *16 schools at the beginning, 2 schools were unable to participate after randomization | IG: school-located vaccination program, with three clinic days per school, including information about program, sent home with students, parents did not have to pay for vaccination  CG (TAU): no school-located vaccination program, only contribution of vaccination data to analyse vaccination rates | School-level: not reported  Participant-level: information about vaccination program was distributed to parents at school registration and via packets sent home with students | Vaccinations offered:  Influenza, Tdap, MCV4, HPV - for females only  Consent process:  participation was not required, parents were asked to provide written consent for their child to be vaccinated at school | Eligibility for study: N=3,144 students eligible for study participation  Eligibility for vaccination: n=527 (17%) provided signed parental consent form | n=466 (88%) received one or more vaccinations,  1,505 doses delivered  🡪 influenza (n=403), HPV (n=326), MCV4  (n=195), hepatitis A (n=180), Tdap (n=168), varicella (n=92) | Outcomes:  IG: Percentage of total program costs reimbursed through paid claims for vaccine purchase and vaccine administration cost, through billing health insurer.  IG vs. CG: Receipt of ≥ 1 dose of recommended vaccinations of Tdap, MCV4, HPV (for females only) for 1,000 randomly selected students from each group with need for vaccination.  Results:  IG: 41% of total program costs were reimbursed, with 78% of vaccine purchase costs and 14% of vaccine administration costs  IG vs. CG: students in the IG were significantly more likely to have received Tdap, MCV4, and HPV vaccines |
| Davies et al. [28] | Australia, Western Australia and South Australia,  40 high schools, grade 8 (girls), grade 9 (boys)  N=6,965 (12-13 years old)  Multi-center cluster-randomized controlled trial  IG: 21 schools, n=3,806;  CG: 19 schools, n=3,159, Stratified (by government, catholic, and independent sectors and geographical location)  Knowledge questionnaire was applied before HPV-vaccination (first dose) and 6 months later (3^rd^ dose) | IG: educational materials for teachers/school nurses were provided. Training for implementation was offered.  Education for students in schools was offered together with a shared decisional support tool for parents and students. Additional  logistical strategies were applied. For the practical implementation, the involved schools, were provided with educational materials about HPV and HPV vaccination and were advised to use the materials at the time consent forms were provided to the parents. Educational materials consisted of a DVD (18 minutes, 7 chapters covering HPV and vaccination, decision-making process, vaccination day, summary), a magazine, a website, and an app  School based vaccination  CG (TAU): school-based vaccination program | School-level:  schools were recruited via a study invitation letter sent to the school principal with telephone follow-up  by study staff  Participant-level:  not reported | Vaccinations offered:  HPV, 3 doses offered within 6 months  Boys were vaccinated in year 9 as part of the HPV catch-up  programme  IG and CG: School based HPV-vaccination, TAU in Australia | Eligibility for study: 153 schools were eligible for participation 🡪 n=117 were invited 🡪 n=40 agreed to participate (26.1%)  Eligibility for vaccination: no information provided | At least one vaccine dose was given to:  n(IG)=3,277 (86.1%)  n(CG)=2697 (85.4%)  More detailed vaccination uptake rates are supposed to be published in a separate paper | Outcomes:  Student knowledge about HPV vaccination,  psycho-social outcomes, vaccination uptake  Results:  Quantitative findings on knowledge questionnaire administered before vaccination show 65% correct answers in the IG vs. 33% in the CG. 6 months later students in the IG had on average 53% correct answers vs. 32% in the CG (p<.001)  Qualitative findings on knowledge of and attitudes towards HPV and HPV vaccination (case study with focus groups, 12 schools participated): knowledge topics mentioned on IG vs. CG are compared (e.g. transmission of HPV) indicating higher level of knowledge and understanding in the IG  No statistical difference regarding vaccination uptake |
| Esposito et al. [22] | Milan, Italy,  12 secondary schools,  N=917 (11-18 years old)*  Prospective, randomized controlled trial with 1:1:1 randomization at class-level  IG1: n=281  IG2: n=302  CG: n=334  Duration: 1 school year, documentation of questionnaire and vaccination coverage at the beginning and the end of the school year  *Inclusion: unvaccinated against diphtheria, tetanus, pertussis, HPV vaccines;  HPV vaccine was recommended only for females. | IG1: same procedure as CG (see below) + participation in a presentation and access to website dedicated to vaccination  IG2: same procedure as IG1 + face to face lecture from medical experts in class  CG: registration of vaccine coverage and attitude toward vaccination at beginning and end of school year | School-level: not reported  Participant-level: not reported | Vaccinations offered:  Influenza, Tdap booster, MenACYW, HPV - for females only, MenB, MenC, Chickenpox  Written consent of students and both students was required to be eligible to study participation  No school based vaccination but vaccinations were administered in vaccination centers during school days | Eligibility for study: N=4,453 attended the 12 schools 🡪 n=1,710 (38.4%) excluded because they already received one or more of the vaccines recommended for adolescents  eligibility for vaccination:  n=2,743 🡪  n=1,380 (50.3%) excluded because of missing written consent ; n=446 (16.3%) excluded because not aged 11-18 years  917 (33.4%) enrolled subjects 🡪who completed the study at the beginning and at the end of a school year:  N(total)=748 (81,6%)  n(IG1)=224 (79.7%)  n(IG2)=238 (78.8%)  n(CG)=284 (85.0%) | Tdap booster:  n(IG1)=99 (44.2%)  n(IG2)=148 (62.2%)  n(CG)=15 (5.3%)  MenACYW vaccine: n(IG1)=52 (23.2%)  n(IG2)=127 (53.4%)  n(CG)= 3 (1.1%)  HPV vaccine:  n(IG1)=9 (4%)  n(IG2)=18 (7.6%)  n(CG)=17 (6%)  MenB vaccine:  n(IG1)=0 (0%)  n(IG2)=41 (17.2%)  n(CG)=0 (0%)  Others: Number of vaccinated subjects too small | Outcomes:  Vaccination coverage,  knowledge and attitudes towards infectious diseases and vaccination  Results:  Significant increase of vaccination coverage for Tdap and MenACYW vaccine in IG1 and IG2, with better results for IG2. There was also a significant increase for MenB vaccine in IG2.  For IG2 there was a significant increase in overall knowledge regarding vaccination and diseases and reduced the fear of vaccines. |
| Forster et al. [29] | London, England, 6 secondary schools, grade 8 from three boroughs N=575 (girls, 12-13 years old, parents)  Equal-allocation, two-arm cluster-randomized controlled trial  IG: 3 schools, n(girls)=255, n(parents)=59;  CG: 3 schools, n(girls)=320, n(parents)=35 | IG(incentive): Students received standard invitation and were told by their tutor and in written form that they would be eligible for a prize draw to win a 50£ voucher, if their signed consent-form is returned (with or without granted consent)  CG: girls were provided with an information leaflet about the HPV vaccine and a consent form from the school to deliver to their parents and return before prescribed date | School-level: all schools in participating boroughs were initially approached via email and then by telephone  Participant-level: letter distributed by the schools, IG letter included a prize draw (incentive). Parental questionnaire via pre-stamped/pre-addressed by mail | Vaccinations offered: HPV  IG and CG:  Within a week to vaccination days, teachers asked girls to complete questionnaire within school hours, parents were sent pre-stamped and pre-addressed questionnaires | Eligibility for study: 6 schools assessed for eligibility 🡪 n=9 (15%) agreed to participate 🡪 6 (10%) completed trial  Eligibility for vaccination:  n(IG)=195 (76.5%), n(CG)=196 (61%) of the girls provided a signed parental consent form,  🡪 returned but not signed:  n(IG)=27 (10.6%)  n(CG)=19 (5.9%) | IG: HPV vaccination rate 15% higher than in CG, no absolute numbers were provided. | Outcome: feasibility of future RCT  (Participation rates, data quality and completeness) proof-of-concept-evidence of intervention effect (consent form return rates, vaccination uptake and any unintended consequences of intervention)  Results:  For participating schools, incentive intervention to improve HPV vaccination uptake is feasible as provided consent forms and HPV vaccinations delivered were higher for the IG (+20% consent forms provided, +15% HPV doses delivered). |
| Grandahl et al. [13] | Sweden, 18 upper secondary school students N=741 aged 16 years  Cluster-randomized controlled trial  IG: 8 schools, n=390;  CG: 10 schools, n=351, randomization at school level + randomized selection of classes within schools  Questionnaire applied at baseline and after 3 months | The intervention was included in the general 1h face-to-face health interview performed by the school nurse  IG: information on sexual health including information regarding HPV (general facts about the virus, transmission, what HPV can cause, risk factors, HPV prevention, where to receive the vaccination free of charge). Information provision was carried out with the use of a specially designed flipcharts with pictures and brief information. Finally, girls received a leaflet (12 pages, including similar information, a HPV quiz, links to the national youth clinic, homepage of university and contacts to authors)  After follow-up questionnaire, IG received condoms  CG: general information, including sexual health | School-level: as school nurses (already working at school) implemented the intervention schools were recruited through their school heads and at a national school health conference  Participant-level: school nurse recruited students in randomized school classes | Vaccination offered: HPV  Girls >12 years and young women are offered the vaccine in the  catch-up programme administered in the  primary care setting | Eligibility for study:  59 upper secondary schools were eligible and approached 🡪 18 were included (20.2%)  Eligibility for vaccination: at school, 2,883 students were eligible 🡪  exclusion: not allocated (n=1659)*^,^ not meeting inclusion criteria (n=388), sick-listed (n=13)🡪 n=832 invited to participate, n=81 declined (9.7%) 🡪 final inclusion of 751 students 🡪 10 students were lost on follow-up n=741 were analysed– all of them provided consent for study participation.  Total amount of consent for vaccination was not reported (One girl who wanted to get vaccinated did not get consent from her parents)  *Despite randomization not allocated, unclear procedure | 15 girls and one boy were vaccinated, 🡪 specific numbers only reported for girls; in the IG n=126 (52.5%) were vaccinated before intervention and 141 (59%) afterwards,  n=68 remained unvaccinated (28.5%). In the CG no vaccination took place during the intervention, n=47 (32.4%) remained unvaccinated.  In total (CG & IG) 35 girls did not know their vaccination status (9%) | Outcome: Intention to use condom with a new partner, beliefs about primary prevention of HPV (Health Belief Model [HBM,[5]] parameters: susceptibility, severity of HPV and barriers of HPV vaccination), vaccination behavior  IG reported significantly higher intention to use condom (p=0.004) but no significant difference regarding the actual use. A significant effect on HBM total score (p=0.003). Influence on the HBM parameters susceptibility and severity significant (p<0001). Girls in the intervention group chose to have themselves vaccinated significantly higher in IG 🡪 increase of vaccination coverage to 59% (+7.5%). |
| Humiston et al. [10] | USA, Monroe County New York, 31 schools, kindergarten through grade 5, N=12,490 students  Community based randomized controlled trial, stratified for urban vs. suburban  IG (suburban, year 1):  n =4,005  IG(suburban, year 2):  n=4,208  CG(suburban, year 1):  n=1,476  CG(suburban, year 2):  n=1,760  IG(urban, year 1):  n=4,356  IG(urban, year 2):  n=4,273  CG(urban, year 1):  n=2,653  CG(urban, year 2):  n=2,635  Duration: 2 years | IG: offer to vaccinate during a normal school day, high and low-intensity parental notification (originally two IG groups, which were combined due to no significant differences regarding vaccination uptake)  CG: no action, no vaccination at schools (TAU), only data provision | School-based: school district superintendents were asked to participate  Participant-level: parental notification through pre-recorded telephone messages, a project website, and print materials sent home with students or mailed to students’ homes either with report cards or separately; materials included vaccine information statements and blank consent forms sent through students backpacks 6-8 weeks before vaccine day, reminder 3 weeks before vaccine day, logistical coordination team collected and reviewed materials regarding completeness and contacted parents for missing information | Vaccinations offered: Influenza  Only school based vaccination for IG  Vaccination status of both groups registered by state immunization information system | Eligibility for study: 6 districts agreed to participate 🡪 9 out of 18 suburban schools participated, 18 of 40 urban schools agreed to participate (45%) 🡪 on a participant level all allocated data was included in final analysis (100%)  Eligibility for vaccination: not reported | Apart from the sample size, no absolute numbers were reported. Uncertain how many parents provided signed consent form (see N – eligibility).  Relative numbers for vaccination coverage:  suburban schools 🡪  prior to program:  IG 29% vs. CG 30%;  Year 1:  IG 47% vs. CG 36%;  Year 2:  IG 52% vs. CG 36%  urban schools 🡪  prior to program:  IG 22% vs. CG 24%;  Year 1:  IG 36% vs. CG 26%  Year 2:  IG 31% vs. CG 25% | Outcome: receipt of seasonal  influenza vaccination  IG vs. CG: OR of 1.6 for receipt of vaccination |
| Rickert et al. [18] | USA, Texas, parents/their kids, which visit a school with a SBHCs* (school based health center: 3 middle schools and 2 high schools)  N=445 (inclusion criteria: with son/daughter 11-15 years old, not received ≥1 dose of HPV vaccine)  Randomized controlled study, 2-level intervention, 2x2 design  n(IG1)=116, n(IG2)=109, n(IG3)=106, n(IG4)=114  *SBHC=alternative primary care setting, part of Teen Health Center (THC).  The THC is a nonprofit organization that works in collaboration University of Texas, Medical Branch (UTMB), to provide health care services, including  immunizations, to residents ages 0 to 21 | Delivery of health message intervention:  IG1: One-sided message (supportive argument) /no RQ  IG2: RQ (rhetorical question: e.g. “Do you want to protect your daughter from cervical cancer?”) and one-sided message  IG3: two-sided message (concerns + supportive=rebutting) /no RQ  IG4: RQ + two-sided message  (incentive 40$ voucher) | School-level: not reported  Participant-level:  CATI (computer assisted telephone interview in English or Spanish), CATI- participants with interest to vaccinate their child, were sent a vaccine information sheet and consent form (to vaccine their kids in the SBHC) in stamped self-addressed envelopes. Parents, who did not return the form, were called 2 weeks later and materials were sent again | Vaccination offered: HPV  For SBHC parents eligible for the study had provided general written consent 🡪 obtaining additional permission for specific vaccination and vaccine information sheet were part of SOP.  Kids with signed parental consent form were scheduled at their respective SBHC for vaccination  Uptake-rate was documented and checked with medical records of SBHC, study team was unable to check on private practitioners or state registry, this rate remains unregistered | Eligibility for study: at the SBHC 1,285 kids were eligible, because participation in SBHC 🡪 additional contact was required for additional consent (HPV) 🡪 N=1,039 (80.6%) were reached by phone 🡪 n=492 (38.3%) were randomized 🡪 n=489 (38.1%) completed interview 🡪 n=445 (34,6%) were finally included (exclusion criteria: already received HPV vaccinations)  Eligibility for vaccination: Of the 1,039 kids/parents n=232 refused to participate (22.3%). Among the final sample, 42.5% (n=189) of parents indicated  that they wanted their son or daughter immunized with  HPV4. Most of the remaining parents (51.4%; n=229) were unsure, with a small proportion (6.1%; n=27) indicating  that they were never going to immunize their teen. | Before intervention 0 students had received a vaccination (see exclusion criteria)  N=151 (of the final sample received their first dose HPV (33.9%) 🡪 n=123 returned for the second dose 🡪 n=101 returned for the third dose | Outcome: Effect of brief health messaging: on parent’s intention to vaccinate their kids, first dose uptake of HPV vaccine, series completion of HPV vaccine  Results: 57.1% of the parents who reported intention to vaccinate, got their child a first dose HPV4 (n=108)  RQ was significantly associated with an intention to vaccinate (RR=1.45), but not with the first uptake (RR=1.15) Message sidedness had no effect on either intention or first dose uptake.  Neither RQ or sidedness was associated with return for a second dose or series completion.  Compared to non-vaccinated adolescents, those who received first dose were younger (13.1 vs. 13.7 years, p <.001) and more likely Hispanic than non-Hispanic (p =.04);  First dose uptake did not differ by parental age, marital stage, educational level, gender of parent interviewed or self-reported insurance status |
| Skinner et al. [30] | Australia, Melbourne, metropolitan secondary schools, 135 schools, grade 7, N=17,411 students  Randomized controlled trial, stratified by metropolitan region (north, south, east or west), school sector (government or independent), co-educational or single sex schools and postcode (indicator of socio-economic status)  IG: 66 schools (n=7,588)  CG: 69 schools (n=9,823) | IG (TAU + hepatitis B education/ promotion kit):  TAU comprised standard state government student and parent information brochures; hepatitis B education comprised 4 lessons, fact sheets, information videos, questions to engage an adolescent audience, small group discussion, internet research 🡪 broadened with activities in the school and the local environment; parental involvement (see recruitment procedure), reminder (posters, stickers and chart monitoring on number of consent forms provided so far) 🡪 educational unit carried out by teachers, they were offered training  CG (TAU): TAU comprised standard state government student and parent information brochures | School-level: not reported  Participant-level: parents were accessed through homework assignment and the composing and distribution of student newsletter articles and letters to the local press | Vaccination offered: hepatitis B  School-based vaccination offered by the local council  Data for vaccination uptake was obtained from the council, this included previously vaccinated students, students vaccinated at school based sessions and those vaccinated at government catch-up sessions. Private provider/ general practitioner vaccinated students were not sampled | Eligibility for study:  180 schools were eligible for participation🡪 n=135 agreed to participate (75%), including 24 schools that were recruited for replacement  Eligibility for vaccination: not reported | No absolute numbers on signed parental consent (eligibility for vaccination) and previous vaccination was reported.  81% of the total samples received their first dose HPV🡪 for the second and third dose the relative rate dropped by 2-3% 🡪 76% completed the course | Outcome: primary outcome was vaccination uptake (mean school level);  Secondary outcomes were gain in knowledge about hepatitis B and change in attitude about hepatitis B (13 schools were included in this secondary analysis)  Results: vaccination rate did not change but significant modifiers for a higher uptake were gender (female), type of school (lower non-English speaking background), class size (larger), SES (high); IG schools with a better implementation of the kit had a higher increase of the vaccination rate (trend); significantly greater knowledge in the IG |
| Tull et al. [20] | Australia, Victoria, 31 schools, majority in a metropolitan area (87.32%) and state government schools (72.25%), grade 7, N=4,386  Randomized controlled trial with 1:1:1 allocation within each school, including a follow-up vaccination (not based at schools) 🡪 inclusion criteria before randomization: parental consent to vaccination  n(IG1)=1,442 n(IG2)=1,418  n(CG)=1,526 | Short message service (SMS) reminders to parents  IG1: Motivational SMS  IG2: Self-regulatory SMS  CG: TAU, comprised regular school based vaccination offer but no reminder | School-level: Victorian Department of Health invited government immunization providers, which are responsible for school based vaccination, to participate in the study.  Participant level: besides the SMS for the IGs nothing additional was reported | Vaccination offered: HPV (focus on dose 3 regarding vaccination schedule, but also dose 1 and 2 were delivered if needed)  Consent process: parental consent was initial condition to be randomized, no further information  IG and CG: School based HPV-vaccination, TAU in Australia, additional end-of-year catch-up sessions (offered and based at the local immunization providers offices) | Eligibility for study: from 12 local government immunization providers in Victoria, 7 agreed to participate representing n=108 schools 🡪  n=31 schools met inclusion criteria (28.70%) and were included  Eligibility for vaccination: n=5,479 enrolled in these schools 🡪 n=4,386 had consent to receive HPV vaccine (80.1%) | No absolute numbers reported, only relative increase in vaccination rate  Vaccination rate before study participation: not reported  Any dose of the HPV vaccine received:  Day of school visit: IG1=88.35%, IG2=89.00%, CG=85.71%  Follow-up:  IG1=91.12%, IG2=90.97%, CG=88.66% | Outcome: vaccination uptake  Results: SMS reminders to parents/guardians lead to greater uptake of the HPV vaccine in adolescents. This effect was observed whether a motivational or self-regulatory message framework was used.  The effect was identically observed in boys’ and girls’ vaccination uptake. |
| Underwood et al. [21] | Georgia, USA,  2,135 students from 11 middle and high schools,  Registration of vaccine coverage before and after two consecutive school years.  Randomization to:  CG: N=777  IG1: N=668  IG2: N=690 | CG: no intervention (TAU)  IG1: educational intervention for parents only (brochures mailed home)  IG2: multicomponent intervention for parents and adolescents (educational brochures for parents mailed home, vaccine-focused curriculum for adolescents presented by science teachers) | Not reported | Vaccinations offered:  Tdap booster, MenACYW, HPV  Vaccination status of all groups was established before and after two school years from the state immunization registry. | Eligibility for study: N=4,877 students selected  N=1,742 excluded because no match to school records  N=618 excluded because no match to state immunization registry records  N=378 excluded because vaccines up to date  N=2,135 participated  No other inclusion criteria reported | Number of adolescents who received at  least one vaccine after intervention  CG=307 (39.5%)  IG1=309 (46.3%)  IG2=346 (50.1%)  Coverage before intervention  Tdap  CG=348 (44.8%) IG1=403 (60.3%) IG2=372 (53.9%)  MenACWY  CG=261 (33.6%) IG1=242 (36.2%) IG2=224 (32.5%)  HPV (at least one dose)  CG=63 (8.1%)  IG1=45 (6.7%)  IG2=45 (6.5%)  Coverage after intervention  (Excludes students who were already up to date)  Tdap  CG=268 (34.5%) IG1=165 (24.7%) IG2=188 (27.3%)  MenACWY  CG=293 (37.7%) IG1=235 (35.2%) IG2=255 (37.0%)  HPV (at least one dose)  CG=203 (26.1%) IG1=180 (27.0%) IG2=168 (24.4%) | Outcomes:  Vaccination coverage  Results:  Adolescents in IG2 were more likely to receive at least one vaccine during the study than those in CG (OR: 1.4; CI: 1.1-2.0) and those in IG1 (OR: 1.4; 95% CI: 1.1-1.7) |

CATI=Computer-assisted telephone interviews; CG=Control group; HIV=Human Immunodeficiency Virus; HPV=(vaccination against) Human papillomavirus; IG=Intervention group; MCV4=Vaccination against meningococcal disease, MenACYW=Vaccination against conjugated meningococcal ACYW; MenB=Vaccination against meningococcal B; PAP-test=Histological investigation of cervix; SMS=Short message service; STI=Sexual transmitted disease; TAU=Treatment as usual, Tdap=Vaccination against tetanus, diphtheria and pertussis, THC=Teen Health Center.

*Note:* PubMed search key words were “vaccination”, AND “school”, AND/OR “intervention”, AND/OR “randomized controlled trial”. Similar articles, cited articles, “cited by” articles. Identification of school-based educational vaccination interventions, to inform the pilot study only studies that offered vaccinations for students on-spot were included, to focus on strategies addressing recruitment, eligibility and vaccinations delivered. Articles published from the year 2000 onwards were included. As part of the review process, the PubMed search was updated on 18.03.2021, for the period of 2019-2021, with the addition of NOT "COVID". As part of the review process, the PubMed search was updated on 23.09.2021.

## Appendix 2

## The Prevention Bus

The Prevention bus consists of a former public bus rebuilt in 2016 into a doctors’ office. The bus is divided into three sections (see Figure 1), each of which is separated by doors. The front area was primarily used as a waiting area. Furthermore, vaccination education could also be carried out in this area. In the second area vaccinations were prepared (vaccine and injection materials) and moreover, vaccinations were carried out in case several students had to be vaccinated at the same time. In this area, there are also located refrigeration facilities for the vaccine as well as an injection drop-off area. The back part of the bus is equipped according to a fully furnished treatment room. Here education and vaccinations were carried out primarily, students could also lie down if necessary.

[INSERT FIGURE 1 APPENDIX ABOUT HERE]

**Fig. 1** Room division in the Prevention Bus, graphic source: Deutsche Bahn AG

[INSERT FIGURE 2 APPENDIX ABOUT HERE]

**Fig. 2** The prevention bus before a school visit in Berlin, picture source: André Solarek, Charité

## Type of schools

With regard to the type of school, a reference specific to Germany's school system is to be given in order to facilitate comparability for other studies. German upper secondary schools, include a range of different high school types. All secondary schools in Berlin start with the 7th school year. Junior high schools are attended until the 10th school year and students can start vocational training once they have graduated with a degree from this school. Junior-senior high schools are structured similarly to junior high schools, but optionally the school can be attended up to 12th or 13th grade and a degree can be obtained qualifying for university attendance. Academic high schools are exclusively attended with the aim of obtaining a university qualifying degree and last until the 12th or 13th school year. The vast majority of these schools in Germany are public schools, including all those that participated in this study. Vocational schools are more diverse (content, age) and teach vocational trainees the contents determined by the curriculum of the respective location during their vocational training. In vocational schools, young people who are undergoing vocational training (e.g., as educators, heating installers, car mechanics, hairdressers, etc.) receive further education in addition to practical training in the company in accordance with the respective curriculum of their training center. Some vocational schools offer the additional option of obtaining a degree qualifying for university attendance.

## Participant recruitment and informed consent

School administrations from selected schools were contacted in advance by letter and email and asked whether they wanted to participate. When an initial interest had been expressed, further questions were clarified by telephone or within a personal appointment at the respective school.

If the school administration had agreed to participate, parents and students were informed one week in advance about the Prevention Bus project with an information leaflet and the consent form that had to be signed for vaccination. Within the leaflet parents and students were also informed that the answering of the questionnaire would be completely anonymous.

On-site, all of the inclusion criteria school classes (9-11) were addressed with one of the intervention conditions. In this context, the questionnaire was also conducted. Both were asked on a voluntary basis. The completion of the questionnaire was anonymous.

For immunization, students could be included if they a) brought their vaccination card and b) the vaccination consent form was signed. All minors had to present a consent form signed by their parents. The parents were asked to complete and hand the documents back to their children who were to bring cards along to school on the day of the intervention. Parental consent forms included a checklist of health related conditions that needed to be met to be eligible for vaccination (e.g., reporting whether the child has had a high fever in the past 14 days, has a coagulation disorder or has had allergic reactions to previous vaccinations). The same checklist was performed again with all students who chose to be vaccinated, as part of vaccination education, before vaccinations could be carried out.

By law in Germany it is not required that parents sign a consent form for vaccination if adolescents are still under age but older than 16 years. Since vaccination is a medical intervention, it was decided that a parental signature was required for all minors receiving vaccination in the context of the study. Additionally, for students under the age of 15, written parental consent was reconfirmed by phone with the parent. Nevertheless, the consent to vaccination of the students was the final mandatory requirement to carry out a vaccination. If minors did not have signed parental consent on the day of vaccination but wanted to be vaccinated, this was a possible incentive to approach parents again and get vaccinated on a later day.

On the day of the on-site intervention all vaccine recipients, including minors, were educated and asked to sign a consent form for vaccination themselves. In case of missing vaccination documents on the day of the intervention, these could be presented during the course of the on-site week and necessary vaccinations could be carried out at any time in the bus on the following days.

## Semistructured interview (ad-hoc translation)

**Table 2** Questions addressed to students regarding the Educational Class Condition and vaccination- process

| Date___________  School type___________  School grade___________ |
| --- |
| How did you like the presentation? |
| What exactly did you like about it? |
| What exactly did you not like? |
| Did you think the presentation was either too long or too short? |
| Were there topics that you found especially interesting? |
| Do you feel like a specific topic was missing? |
| Annotation page 1: |
| How did you like the example of Luan in the physician’s office? |
| How did you feel about the fact that images of the diseases were shown? |
| Were the physician’s explanations easily understandable for you? |
| Which presented information about vaccinations surprised you the most? Which information was new to you? |
| In case you have a need for vaccination, would you currently decide to get vaccinated? (If need for vaccination is known, ask directly) |
| Why? (Why do you think that vaccinations are important/what might be reasons against a vaccine?) |
| Was your opinion regarding this topic different before listening to the presentation? |
| Annotation page 2: |
| Did the vaccination card check distract or disturb you during class? |
| Do you have any other suggestions/wishes/tips for us? |
| Further comments: |

## Scales

**Vaccination-related knowledge (ad-hoc translation)**

There was an adjustment of the knowledge items used, since in week one (school 1) the physicians on-site reported frequent comprehension problems experienced by some students with regard to Item 5 (*bacteria*). Accordingly, the opportunity was taken to introduce two further items and delete the one causing comprehension problems. Both newly added items addressed complications of infectious diseases. The first newly introduced item covered the complication of male infertility due to mumps and the second new item dealt with malformations of embryos during pregnancy due to rubella. In order to balance possible bias in the response caused by gender, these two items were added successively.

**Table 3** Vaccination related knowledge items applied in the pilot study

| Item 1  C*hildhood disease* | Which groups of people can get infected with the so-called childhood diseases? | □ Children  □ Children and their parents  □ People of all ages  □ Only people who have contact with children |
| --- | --- | --- |
| Item 2  *Herd immunity* | What does herd immunity mean? | □ People who live together in an apartment protect each other from illnesses.  □ Unvaccinated people are protected by the vaccination protection of other vaccinated people.  □ Newborns are protected after birth by the vaccination protection of the mother against illnesses.  □ People who live together in an apartment are protected if at least one person is vaccinated. |
| Item 3  *Immunization* | What is the ideal timing for vaccination in order to achieve the best possible protection? | □ After one had the illness already, in order not to fall ill again  □ If you have had contact with a sick person to avoid infection  □ Preventive if you are healthy  □ If you are already ill to prevent a dramatic course of the disease |
| Item 4  *Measles outbreak* | How can the spreading of measles among the population be prevented in the long term? | □ Closure of public institutions  □ A single vaccination  □ If at least 95% of the population have sufficient vaccination protection  □ Cessation of tourist traffic |
| Item 5  *Bacteria* | What is not an entry gate for pathogens? | □ Conjunctival membrane in the eye  □ Mucosa of the respiratory tract  □ gastrointestinal tract  □ Intact skin |
| Item 6  *Infertility* | Which of the following diseases can cause infertility in boys? | □ Poliomyelitis (poliomyelitis)  □ Mumps  □ Rubella  □ Hepatitis B |
| Item 7  *Pregnancy malformation* | What disease can cause malformations of the baby if a woman becomes ill during pregnancy? | □ Measles  □ Mumps  □ Rubella  □ Hepatitis B |

**Perceived self-efficacy (selected items, European Health Literacy Survey Questionnaire [HLS-EU Q47 [10])**

**Table 4** Perceived self-efficacy items applied in the pilot study

| On a scale from very easy to very difficult, how easy would you say it is to: … | |
| --- | --- |
| Item 1 | understand why you need vaccinations? |
| Item 2 | judge which vaccinations you may need? |
| Item 3 | judge if the information on health risks in the media is reliable? |
| Item 4 | decide how you can protect yourself from illness based on advice from family and friends? |
| Item 5 | decide how you can protect yourself from illness based on information in the media? |
|  |  |
| Rating for all items | 1 – Very difficult; 2 – Difficult; 3 – Easy; 4 – Very easy |
